# Histone variant MacroH2A1 is downregulated in prostate cancer and influences malignant cell phenotype

**DOI:** 10.1186/s12935-019-0835-9

**Published:** 2019-04-29

**Authors:** Tânia Soraia Vieira-Silva, Sara Monteiro-Reis, Daniela Barros-Silva, João Ramalho-Carvalho, Inês Graça, Isa Carneiro, Ana Teresa Martins, Jorge Oliveira, Luis Antunes, Sarah Hurtado-Bagès, Marcus Buschbeck, Rui Henrique, Carmen Jerónimo

**Affiliations:** 1 Cancer Biology & Epigenetics Group, Research Center (CI-IPOP), Portuguese Oncology Institute of Porto (IPO Porto), Research Center-LAB 3, F Bdg., 1st Floor, Rua Dr. António Bernardino de Almeida, 4200-072 Porto, Portugal; 2 Department of Pathology, Portuguese Oncology Institute of Porto (IPO Porto), Rua Dr. António Bernardino de Almeida, 4200-072 Porto, Portugal; 3 Department of Urology, Portuguese Oncology Institute of Porto (IPO Porto), Rua Dr. António Bernardino de Almeida, 4200-072 Porto, Portugal; 4 Department of Epidemiology, Portuguese Oncology Institute of Porto (IPO Porto), Rua Dr. António Bernardino de Almeida, 4200-072 Porto, Portugal; 5 Josep Carreras Leukaemia Research Institute (IJC), Campus ICO-Germans Trias i Pujol, Campus Can Ruti, 08916 Badalona, Spain; 6 Program for Predictive and Personalized Medicine of Cancer, Germans Trias i Pujol Research Institute (PMPPC-IGTP), Barcelona, Spain; 7 Department of Pathology and Molecular Immunology, Institute of Biomedical Sciences Abel Salazar (ICBAS), University of Porto, Porto, Portugal

**Keywords:** Prostate cancer, Histone variants, MacroH2A1 isoforms, Splicing regulators, Tumor suppressor

## Abstract

**Background:**

Prostate cancer (PCa), a major cause of cancer-related morbidity and mortality worldwide and mostly asymptomatic at earliest stages, is characterized by disruption of genetic and epigenetic balance. A better understanding of how those mechanisms orchestrate disease might improve diagnostic and prognostic tools, allowing for improvements in treatment efficacy. Replacement of canonical histones, an epigenetic mechanism, is highly conserved among species and altered expression of histones variants (e.g., MacroH2A1) has been associated with tumorigenesis. H2AFY gene encodes two isoforms of H2A histone variant MacroH2A1: MacroH2A1.1 and MacroH2A1.2. Specifically, MacroH2A1.1 isoform inhibits cell proliferation and promotes cellular differentiation. Because the contribution of this histone variant to carcinogenesis has been reported in several cancer types, but not for PCa, we aimed to investigate the contribution of MacroH2A1 for prostate carcinogenesis.

**Methods:**

MacroH2A1, MacroH2A1.1 and MacroH2A1.2 isoforms and the corresponding splicing regulators transcript levels were evaluated by RT-qPCR, in a tissue cohort composed by PCa, prostatic intraepithelial neoplasia (PIN) and normal prostate cases. Knockdown for MacroH2A1 and MacroH2A1.1 was performed through lentiviral transduction in DU145 cells, and MacroH2A1.1 overexpression was achieved in LNCaP cells by plasmid transfection, followed by functional assays. Biological and/or experimental replicates were performed when necessary, and specific statistical tests were applied to perform data analysis.

**Results:**

MacroH2A1.1 transcript levels were downregulated in PIN and primary PCa compared to normal prostate tissues. The same was found for QKI, a MacroH2A1.1’s splicing regulator. Moreover, lower MacroH2A1.1 and QKI expression levels associated with less differentiated tumors (Gleason score ≥ 7). Interestingly, MacroH2A1.1, but more impressively DDX17 (AUC = 0.93; p < 0.0001) and QKI (AUC = 0.94; p < 0.0001), accurately discriminated cancerous from noncancerous prostate tissues. Furthermore, in PCa cell lines, total MacroH2A1 knockdown augmented malignant features, whereas MacroH2A1.1 overexpression impressively attenuated the malignant phenotype.

**Conclusions:**

Overall, our data, derived from primary PCa tissues and cell lines, anticipate a tumor suppressive role for MacroH2A1, particularly for the MacroH2A1.1 isoform, in prostate carcinogenesis.

**Electronic supplementary material:**

The online version of this article (10.1186/s12935-019-0835-9) contains supplementary material, which is available to authorized users.

## Background

Prostate cancer (PCa) is the most common non-cutaneous malignancy in men and a major cause of cancer-related morbidity and mortality worldwide [1]. Characteristically asymptomatic at its earliest stages, when curative-intended therapy is most likely to be successful, PCa is characterized by a broad range of alterations in both genetic and epigenetic level [2, 3].

Among epigenetic mechanisms deregulated in cancer, a relevance of changes in histone variants that can replace canonical histones (nowadays referred to as replication-coupled histones) has just started to emerge while still being poorly understood [4, 5]. Unlike canonical histones, variants display time-based incorporation at the nucleosomes, are tissue-specific and DNA replication-independent [6]. The H2A family of histones comprises structurally diverse variants, including H2A.X, H2A.Z, MacroH2A1, H2A.Bb, characterized by distinct length, sequence and genome distribution [7, 8]. Deregulation in H2A variants have been previously implicated in cancer [4]. The *H2AFY* gene encodes for MacroH2A1, a histone variant frequently found in repressed chromatin such as the inactive X chromosome in female cells [9, 10]. Moreover, a subset of genes positively regulated by MacroH2A1 has also been reported [11–13]. MacroH2A1 is present in Senescence-Associated Heterochromatic Foci [14], as well as on genes involved in cell cycle [15], pluripotency [16] and development [10, 17]. Additionally, two isoforms of MacroH2A1—MacroH2A1.1 and MacroH2A1.2—result from the substitution of a single exon [4, 18]. MacroH2A1.1 is preferentially expressed over 1.2 in differentiated cells whereas MacroH2A1.2 is more expressed in proliferative cells [19, 20]. The ratio of MacroH2A1 isoforms expression levels is regulated by pre-mRNA splicing regulators. Indeed, without altering global MacroH2A1 expression, QKI promotes MacroH2A1.1 expression, whilst MacroH2A1.2 expression is promoted by RNA helicases DDX5/DDX17 [21, 22].

Alterations in the expression of total MacroH2A1 or its isoforms [19] has been observed in several cancer types, including breast carcinoma [22, 23], melanoma [24], lung carcinoma [25] and colorectal carcinoma [26]. Taken the bulk literature together the MacroH2A1.1 isoform emerges as pleiotropic tumor suppressor, by repressing cell proliferation, migration and invasion, whereas the role of MacroH2A1.2 is largely cancer-type dependent [18, 27].

We have previously demonstrated that the histone H2A variant H2A.Z is upregulated in PCa [28], suggesting that the substitution of canonical histones by variants might be implicated in prostate carcinogenesis. As opposing functions for H2AZ and MacroH2A1 in gene regulation were reported in cancer cells [29], we investigated the role of MacroH2A1.1 and MacroH2A1.2 isoforms in PCa development.

## Materials and methods

### Patients and clinical samples

Tumor samples from 197 Prostate cancer patients and 45 PIN lesions were prospectively collected from radical prostatectomy specimens obtained from 2001 to 2006, at the Portuguese Oncology Institute of Porto, Portugal. Among these, 34 patients had both PIN lesions and PCa samples. Immediately after surgery, specimens were fully sectioned and “twin fragments” were obtained, one for routine histopathological processing and the other was frozen and stored at − 80 °C. After full mapping of each prostate in FFPE tissue sections, the index tumor (the tumor nodule in multifocal disease that portrays the most relevant combination of prognostically relevant histo-morphological parameters, i.e., stage/local extension and grade) and PIN lesions were identified. Each PCa case was staged and the corresponding index tumor was scored according to the Gleason grading system [30]. Then, a frozen fragment corresponding to the index tumor and another corresponding to the PIN lesion were selected for RNA extraction. For that purpose, frozen sections were cut, and H&E stained for allowing for confirmation of the presence PIN lesions and index tumor, including Gleason score representability. Then, each tissue fragment was trimmed to maximize the yield of target cells (> 70% of target cells) through serial cutting of thick (10 µm) sections. This procedure was performed by the same experienced uropathologist (RH) and allowed for confirmation that both PIN lesions and tumor areas identified in the frozen sections were representative of the index lesions identified in the routine assessment of the prostatectomy specimen. Relevant clinical data was retrieved from clinical charts. Fifteen samples of MNPT, used as controls, were obtained from the peripheral zone of prostates not harboring PCa, obtained from radical cystoprostatectomies performed for bladder cancer treatment that were submitted to the same procedure as prostate specimens that harbored cancer.

### Prostate cancer cell lines

Benign prostate cell line RWPE-1 and PCa cell lines 22Rv1, LNCaP and VCaP, which are androgen receptor (AR) positive, as well as PCa cell lines DU145 and PC-3, characterized as AR negative, were grown for in vitro assays. RWPE-1 cells were maintained in K-SFM growth medium supplemented with Bovine pituitary extract + Human recombinant epidermal growth factor and 1% penicillin/streptomycin. 22Rv1 and LNCaP cells were grown in RPMI 1640, DU145 and VCaP cells were maintained in MEM and PC-3 cells were grown in 50% RPMI-1640 + 50% F-12 medium (GIBCO^®^). The culture media of PCa cell lines were supplemented with 10% fetal bovine serum and 1% penicillin/streptomycin (GIBCO^®^). Cells were grown in an incubator at 37 °C with 5% CO_2_. All prostate cell lines were tested for *Mycoplasma* spp. contamination (PCR Mycoplasma Detection Set, Clontech Laboratories). Cells later harvested for protein and RNA extraction.

### RNA extraction and quantitative reverse transcription PCR (qRT-PCR)

Samples were homogenized in TRIzol^®^ Reagent (Invitrogen) and the total RNA was extracted using PureLink™ RNA Mini Kit (Invitrogen). All genomic DNA was eliminated with TURBO DNA-free (Ambion, Applied Biosystems), according to manufacturer’s instructions. First strand synthesis was performed using the TransPlex^®^ Whole Transcriptome Amplification Kit (Sigma-Aldrich^®^) and QIAquick PCR Purification Kit (QIAGEN) for purification.

Target genes’ expression was determined using Fast SYBR Green^®^ Gene Expression Assay (Applied Biosystems^®^) and normalized to the expression of the endogenous control β-glucuronidase (*GUSB)*, a housekeeping gene. Primers were *MacroH2A1.1*: forward, 5′-GGCTTCACAGTCCTCTCCAC-3′, and reverse, 5′-GGTGAACGACAGCATCACTG-3′; *MacroH2A1.2*: forward, 5′- GGCTTCACAGTCCTCTCCAC-3′, and reverse, 5′-GGATTGATTATGGCCTCCAC-3′; *MacroH2A1*: forward, 5′-TCCATTGCATTTCCATCCATCGGC-3′, and reverse, 5′-ACACGAAGTAACTGGAGATGGCCT-3′; *QKI*: forward, 5′-ATTAAACGGTCCCCTGAAGC-3′, and reverse, 5′-ATCAACAGCCCAAGTGTGAC-3′; *DDX5*: forward, 5′-GTAGCTCAGACTGGATCTGG-3′, and reverse, 5′-TCTCTAGGAATGGCTGGTGG-3′*DDX17*: forward, 5′-AGAAGTAGCAAGACT GACTCC-3′, and reverse, 5′-CCCCCTCTCACTGTAATCTC-3′; *GUSB*: forward, 5′-CTCATTTGGAATTTTGCCGATT-3′, and reverse, 5′-CCGAGTGAAGATCCCCTTTTTA-3′; and H2AZ forward, 5′-GGGAAGAAAGGACAACAG-3′, and reverse, 5′-CACAGAGATACAGTCCACTGG-3′. RNA levels were determined by the standard curve method. All samples were analyzed in triplicate in a 7500 Real-Time PCR system (Applied Biosystems^®^), and the mean value was used for data analysis.

### Transfection of cell lines

DU145 cells, which showed the highest MacroH2A1.1 expression levels, were selected for knocking-down assays. Thus, cells were transiently transfected with SMARTpool: siGENOME H2AFY siRNA (Dharmacon) to knockdown MacroH2A1, and for MacroH2A1.1, cells were transiently transfected with a previously published siRNA pool at 25 nM, and a siRNA negative control served as control in all experiments [22]. Oligofectamine™ reagent (Invitrogen, USA) was used for transfection under conditions indicated by the manufacturer. Cells were seeded 24 h before transfection, according to the purpose: for MTT assay, 4000 cells/well were seeded (96-well plates); apoptosis evaluation 30,000 cells/well were plated (24-well plates); and for RNA and protein, 200,000 cells/well were used (6-well plates). The siRNA transfection was performed only once after cell seeding.

Overexpression of MacroH2A1.1 was achieved in LNCaP cells that depicted the lowest transcript levels. This was performed through pEZ-Lv105 (GeneCopoeiaTM) using FuGENE^®^ HD Transfection Reagent (Promega), following manufacturer’s recommendations. After transfection, stable cell lines transfected with the vector and selected with puromycin dihydrochloride for in vitro assays. The stable cell lines generated where then use to test cell viability (MTT assay, 10,000 cells/well), apoptosis (50,000 cells/well), and for RNA and protein.

In both conditions, protein and RNA extraction was performed in cells harvested at 72 h.

### SDS-PAGE and western blot

Total protein was extracted from cell lines using the Kinexus Lysis Buffer with protease inhibitor (Kinexus Bioinformatics Corporation). Protein concentration was determined using Pierce BCA Protein Assay Kit (Thermo Scientific) following manufacturer’s instructions. Then, 30 μg of total protein were separated by SDS-PAGE, blotted in PVDF membranes (BioRad) and incubated in 5% (w/v) bovine serum albumin (BSA) blocking solution (ChemCruz™) for 1 h. Membranes were probed with antibodies against MacroH2A1.1 (#12,455, Cell Signaling), MacroH2A1.2 (#4827; Cell Signaling), Cleaved PARP (Asp214) (19F4) (#9546, Cell Signaling) or the endogenous control β-actin (Sigma). Western Bright™ ECL-spray (Advansta) was used to develop the blots. Triplicates were performed in all experiments. Relative optical density determination was performed using ImageJ and normalized for the loading control, β-actin.

### Cell viability

Cell viability was assessed in transfected LNCaP and DU145 cells using the 3-(4, 5-dimethylthiazol-2-yl)-2,5-diphenyltetrazolium (MTT; Sigma-Aldrich^®^) assay. The viability assay was performed after cells adhered to the plate and, for DU145 cell line, right before transfection (0 h) and in the subsequent days (24 and 48 h). MTT was added to the cells at 37 °C for 1 h, formazan crystals were dissolved in 100 μL of Dimethyl sulfoxide DMSO (Sigma-Aldrich^®^) and plates shaken for 15 min. Colorimetric quantification was performed in an automated plate reader GloMax^®^-Multi Detection System (Promega) at 560 nm, with a reference filter of 630 nm. The optical density (OD) was directly proportional to the number of viable cells. Three biologically independent experiments and methodological triplicates were done for all experiments.

### Apoptosis

APOPercentage™ kit (Biocolor) was used to evaluate apoptotic levels using a multi-plate GloMax^®^-Multi Detection System (Promega), for OD measurement of the released dye at 550 nm with a reference filter of 620 nm. To normalize the OD measured in the apoptotic test to the cell number, the OD of apoptosis was normalized to the OD of the viability assay. Three biological independent experiments were performed with methodological triplicates for each experiment.

### Statistical analysis

Differences in quantitative expression levels of MacroH2A1 and splicing regulators among MNPT, PIN and PCa were assessed using the non-parametric Kruskal–Wallis test, followed by pairwise comparisons with Mann–Whitney *U*-test, with Bonferroni’s correction. Differences in gene expression between matched PIN and PCa samples were calculated by Wilcoxon Signed Rank test. Spearman’s correlation test was used to evaluate the association between transcript levels of different genes. The associations between expression levels and standard clinicopathological variables (serum PSA levels at diagnosis, Gleason score, histopathological stage) were assessed using the Kruskal–Wallis or Mann–Whitney tests, as appropriate. A receiver operator characteristic (ROC) curve, its area under the curve and respective confidence intervals, were constructed, as described in [30], to assess the performance of MacroH2A1.1 and splicing regulators expression for discriminating PCa from MNPT [31]. To demonstrate that the confidence intervals for the AUC obtained in this series (MNPT = 15 and PCa = 197) are statistically robust, a simulation study was performed (Additional file 1: Data S1). Moreover, for the same genes, biomarker sensitivity, specificity and accuracy parameters were calculated, as well as the positive (LR+) and negative (LR−) likelihood ratios. Regarding this, as the quantitative value of a calculated likelihood ratio is further away from 1 in either direction (> 1 for LR+ and < 1 for LR−), there is increasing utility of a diagnostic test to point toward, or away from, a diagnosis which indicate the value of performing the respective diagnostic tests. Correlation between MacroH2A1, respective isoforms and splicing regulators or H2A.Z expression levels were assessed by Spearman’s correlation coefficient (r).

In cell lines, fold variation differences in transcript and protein levels were determined using One-Way Analysis of Variance (ANOVA), followed by Dunnet’s (post hoc) test for multiple comparisons, or unpaired t-test, as appropriate, comparing all PCa cell lines against RWPE-1 or negative control of transfection.

All tests were two-sided and statistical significance was set at *p *< 0.05. Statistical analysis was performed using GraphPad Prism software for Windows version 5.0 (GraphPad Software Inc., La Jolla, CA, USA).

## Results

### Isoform MacroH2A1.1 is downregulated in primary PCa

**Fig. 1:**
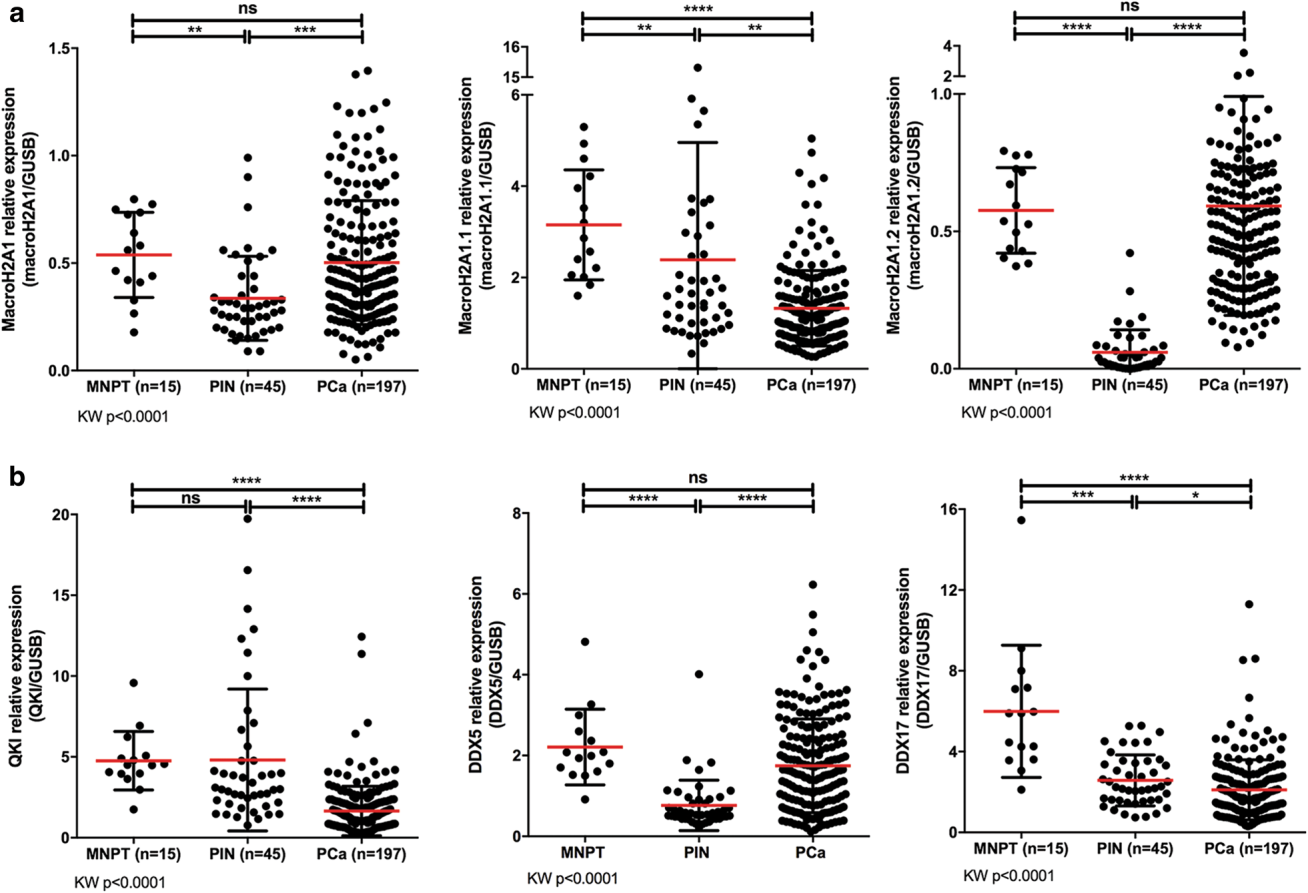
Transcript levels of MacroH2A1 and splicing regulators in prostate tissue samples. **a** Transcriptional status of total MacroH2A1, MacroH2A1.1 and MacroH2A1.2 isoforms in MNPT samples (n = 15), PIN lesions (n = 45) and PCa samples (n = 197), assessed by RT-qPCR, and normalized with GUSB gene. **b** Transcript levels of three splicing regulators of *H2AFY* mRNA—QKI, DDX5 and DDX17—assessed by RT-qPCR, and normalized with GUSB gene. *MNPT* morphologically normal prostate tissue, *PIN* prostatic intraepithelial neoplasia, *PCa* prostate carcinoma, *KW* Kruskal–Wallis test. Inter-group analysis with Kruskal–Wallis test, followed by Dunn’s multiple comparisons test: *p < 0.05, **p < 0.01, ***p < 0.001, ****p < 0.0001, *ns* not significant

Relevant clinical and pathological data of patients included in this study is depicted in Table 1. MacroH2A1, MacroH2A1.1 and MacroH2A1.2 transcript levels were independently assessed in 15 MNPT, 45 PIN and 197 PCa tissue samples (Fig. 1a, Additional file 2: Table S1). Age was not significantly different among the studied groups.

**Table 1:** Clinical and pathological features of subjects in each group of tissue samples

Clinicopathological features	MNPT	PIN	PCa
Number of subjects, n	15	45	197
Age (years)			
Median (range)	64 (45–80)	64 (51–75)	64 (49–75)
PSA levels (ng/mL)			
Median (range)	na	na	8.3 (2.9–23)
Pathological stage			
pT2, n (%)	na	na	110 (55.8)
pT3, n (%)			87 (44.2)
Gleason score [n (%)]			
6	na	na	67 (34.0)
7			115 (58.4)
8			6 (3.0)
9			9 (4.6)

*na* not applicable, *MNPT* morphologically normal prostate tissue, *PIN* prostatic intraepithelial neoplasia, *PCa* prostate carcinoma

MacroH2A1 expression levels did not differ significantly between MNPT and PCa, whereas a significant decrease was observed in PIN (p < 0.001) (Fig. 1a, Additional file 2: Table S1). MacroH2A1.1 expression levels, however, differed significantly among the three groups [Kruskal–Wallis (KW) test, p < 0.001], with the lowest transcript levels found in PCa (p < 0.001 and p = 0.009, compared to MNPT and to PIN, respectively). Indeed, PIN lesions disclosed higher MacroH2A1.1 transcript levels than PCa, in cases in which PIN and matched tumor lesions from the same prostatectomy specimens were available (Additional file 3: Fig. S1). Regarding MacroH2A1.2, significantly lower expression levels were depicted for PIN comparing with MNPT and PCa (p < 0.001), whereas no significant differences were apparent between MNPT and PCa.

Overall, PCa tissues showed higher MacroH2A1.1 (1.33) than MacroH2A1.2 (0.59) relative expression levels, although the highest MacroH2A1.1 levels were observed in PIN lesions (2.39). Conversely, PIN lesions displayed the lowest levels of MacroH2A1.2 (0.06) (Fig. 1a, Additional file 2: Table S1).

### H2AFY splicing regulators and H2AFZ expression in prostate cancer

To explain the altered ratio between *H2AFY* gene splicing variants, MacroH2A1 splicing regulators (QKI, DDX5 and DDX17) transcription levels were also assessed in the same sample sets (Fig. 1b, Additional file 2: Table S1). A statistically significant downregulation of QKI and DDX17 was depicted for PCa (p < 0.001), whereas in PIN, DDX17 expression levels were significantly lower compared to MNPT (p < 0.001). Concerning DDX5 expression, PIN demonstrated the lowest levels (p < 0.001, both for MNPT and PCa) but no significant differences were apparent between MNPT and PCa samples (Fig. 1b, Additional file 2: Table S1).

DDX5 transcript levels showed a stronger positive correlation with MacroH2A1 total expression (ρ = 0.51, p < 0.001), whereas a significant correlation was also found between QKI and MacroH2A1.1 splice variant expression levels (ρ = 0.56, p < 0.001). Splicing regulator DDX17 expression levels did not impressively correlate with any of the MacroH2A1 transcripts (Additional file 2: Table S2). Furthermore, no inverse correlation was found between *H2AFY* and *H2AFZ* transcript levels (Additional file 3: Fig. S2).

### Diagnostic performance of MacroH2A1.1 and splicing regulators transcript levels in prostate tissues

**Fig. 2:**
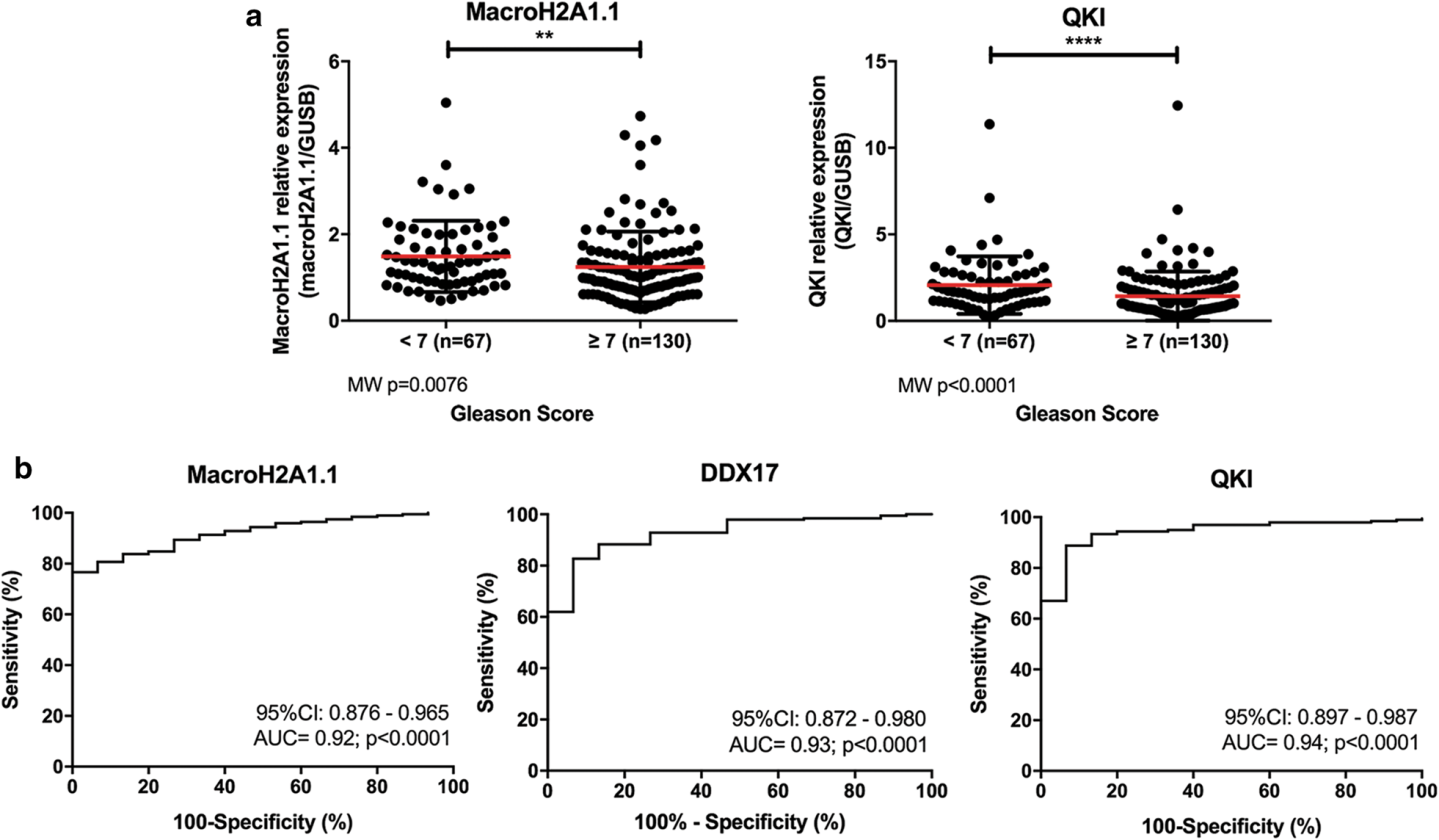
Association of MacroH2A1.1 and QKI transcript levels with clinicopathological parameters and diagnostic performance. **a** Distribution of MacroH2A1.1 and QKI transcript levels in PCa cases, determined by RT-qPCR and normalized with GUSB mRNA levels, according to categorized < 7 (n = 68) and ≥ 7 (n = 129) Gleason score; Mann–Whitney *U* test: **p < 0.01, ****p < 0.0001. **b** ROC curves showing biomarker performance evaluation of MacroH2A1.1 (AUC = 0.92, 95% CI 0.876–0.965, p < 0.0001), DDX17 (AUC = 0.93, 95% CI 0.872–0.980, p < 0.0001), and QKI (AUC = 0.94, 95% CI 0.897–0.987, p < 0.0001) transcript levels as discriminators of prostate cancer (PCa, n = 195) from morphological normal prostate tissue (MNPT, n = 15). *AUC* area under curve, *CI* confidence interval

Regarding associations with clinical-pathological variables, high MacroH2A1.1 expression levels significantly associated with serum PSA levels above 10 ng/mL (p < 0.01, (Additional file 3: Fig. S3). Moreover, MacroH2A1.1 and QKI expression levels significantly associated with Gleason score (Fig. 2a), a clinical parameter of disease progression and reduced differentiation. Indeed, considering a two-tier categorization (Gleason score = 6 vs. Gleason score > 6), higher grade (less differentiated) tumors displayed significantly reduced MacroH2A1.1 (p < 0.01) and QKI (p < 0.001) expression levels. Considering a four-tier system, corresponding to the Gleason scores 6, 7 (3 + 4), 7 (4 + 3) and ≥ 8, differences among the four categories were found both for MacroH2A1.1 and QKI expression (p = 0.0185 and p < 0.0001, respectively; Additional file 3: Fig. S4). Pairwise analysis disclosed significant differences between Gleason scores 6 and 7 (3 + 4) for MacroH2A1.1 and QKI expression (p = 0.0179 and p = 0.0082, respectively), and between Gleason scores 6 and ≥ 8 (p < 0.0001).

Receiver operator characteristic (ROC) curve analysis was performed to assess the ability of MacroH2A1.1, DDX5, DDX17 and QKI expression levels in discriminating PCa from non-cancerous prostate tissues (Fig. 2b and Table 2). The empirical cut-off values were set to maximize sensitivity and specificity. Remarkably, QKI outperformed MacroH2A1.1 and DDX5 and DDX17, displaying 88.8% sensitivity and 93.3% specificity, corresponding to an area under the curve (AUC) of 0.94 (95% IC: 0.897-0.987); p < 0.0001 (Table 2). Moreover, QKI presented a LR+ of 13.25 and a LR− of 0.12 (Table 2). Notwithstanding the limited number of normal tissue samples, a statistical model (1000 simulations) disclosed mean confidence interval range of 0.101, with 25% and 75% percentiles of 0.079 and 0.119. Since the value obtained with the real dataset was 0.089, we may consider that is within the range of expected values (Additional file 1: Data S1).

**Table 2:** Validity estimates for MacroH2A1.1 and QKI expression levels as diagnostic biomarkers for prostate cancer identification

Parameter	MacroH2A1.1 performance	DDX5 performance	DDX17 performance	QKI performance
Sensitivity (%)	80.7	82.7	47.3	88.8
Specificity (%)	93.3	93.3	93.3	93.3
Accuracy (%)	81.6	83.5	50.5	88.7
Positive likelihood ratio (LR+)	12.04	12.34	7.06	13.25
Negative likelihood ratio (LR−)	0.21	0.19	0.56	0.12
AUC (95% IC) p value	0.92 (0.876–0.965) p < 0.0001	0.65 (0.549–0.751) p = 0.0533	0.93 (0.872–0.980) p < 0.0001	0.94 (0.897–0.987) p < 0.0001

### MacroH2A1 phenotypic impact in PCa cell lines

**Fig. 3:**
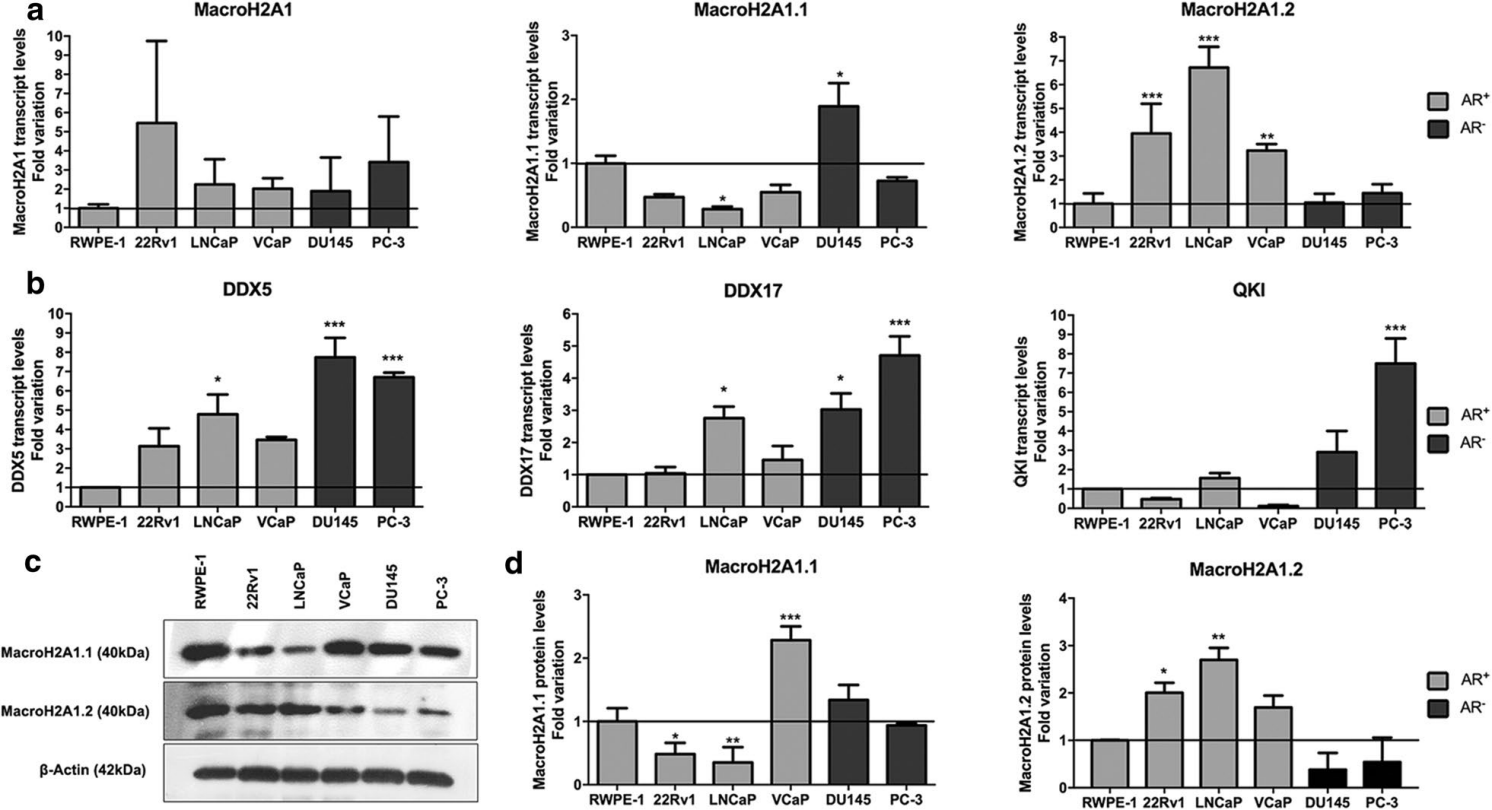
Distribution of MacroH2A1 transcript and protein levels in prostate cancer cell lines. **a** Transcript levels of total MacroH2A1, MacroH2A1.1 and MacroH2A1.2, assessed by RT-qPCR and normalized to GUSB mRNA levels, in androgen-receptor positive RWPE-1 benign prostate and 22Rv1, LNCaP and VCaP cancer cell lines, and in androgen-receptor negative DU145 and PC-3 prostate cancer cell lines. **b** Transcript levels of splicing regulators DDX5, DDX17 and QKI, assessed by RT-qPCR and normalized to GUSB, in androgen positive and negative prostate cell lines. **c** Illustrative images of MacroH2A1 isoforms protein levels in prostate cancer cell lines and in non-malignant prostate cell line RWPE-1. **d** Fold variation of MacroH2A1.1 and MacroH2A1.2 protein levels, normalized to β-actin, and directly compared to benign prostate cell line RWPE-1. (mean ± SD, n = 3). Dunnet’s pairwise multiple comparisons test: *p < 0.05, **p < 0.01, ***p < 0.001

Transcript levels of MacroH2A1, respective isoforms and splicing regulators were assessed by RT-qPCR in five PCa cell lines (22Rv1, LNCaP, VCaP, DU145 and PC-3) and normalized for a benign prostate cell line (RWPE-1) (Fig. 3a, b).

Although 22Rv1 showed the highest MacroH2A1 transcript levels, no significant differences were apparent among PCa cell lines, comparing with RWPE-1. AR negative (AR−) PCa cell line DU145 displayed significantly higher MacroH2A1.1 expression levels than RWPE-1 (p < 0.05), whereas the lowest transcript levels of this variant were depicted for LNCaP, an AR positive (AR+) PCa cell line (p < 0.05). Concerning MacroH2A1.2, all AR+ PCa cell lines (22Rv1, LNCaP and VCaP) displayed significantly higher expression levels than the benign prostate cell line (p < 0.01, for all) whereas AR− cancer lines did not disclose significant differences compared to RWPE-1 (Fig. 3a). As to splicing regulators, among AR+ cell lines, only LNCaP depicted significantly higher *DDX5* and *DDX17* transcript levels than RWPE-1, whereas both AR− cells disclosed significantly higher *DDX5*, *DDX17* and *QKI* mRNA level, cells except for *QKI* in DU145 (p < 0.05, Fig. 3b). Although no significant differences in global MacroH2A1 transcript levels between AR+ and AR− cancer cell lines were found, AR− cells depicted significantly higher MacroH2A1.1 expression levels than AR+ cells, whereas the opposite was found for MacroH2A1.2 (Additional file 3: Fig. S5a). Nonetheless, among splicing regulators, significant differences in splicing regulators expression levels between AR+ and AR− were only found for QKI (Additional file 3: Fig. S5b).

Concerning protein expression, MacroH2A1.1 and MacroH2A1.2 protein levels were also rather variable among cell lines (Fig. 3c, d). MacroH2A1.1 protein expression pattern only differed from transcript levels in VCaP and DU145 cell lines, whereas for MacroH2A1.2, protein levels in PCa cell lines followed the same trend of the transcript. Interestingly, MacroH2A1.2 proteins levels were significantly higher in AR+ vs. AR− PCa cell lines (paralleling the findings at transcript level), whereas no differences were found for MacroH2A1.1 (Additional file 3: Fig. S5c).

**Fig. 4:**
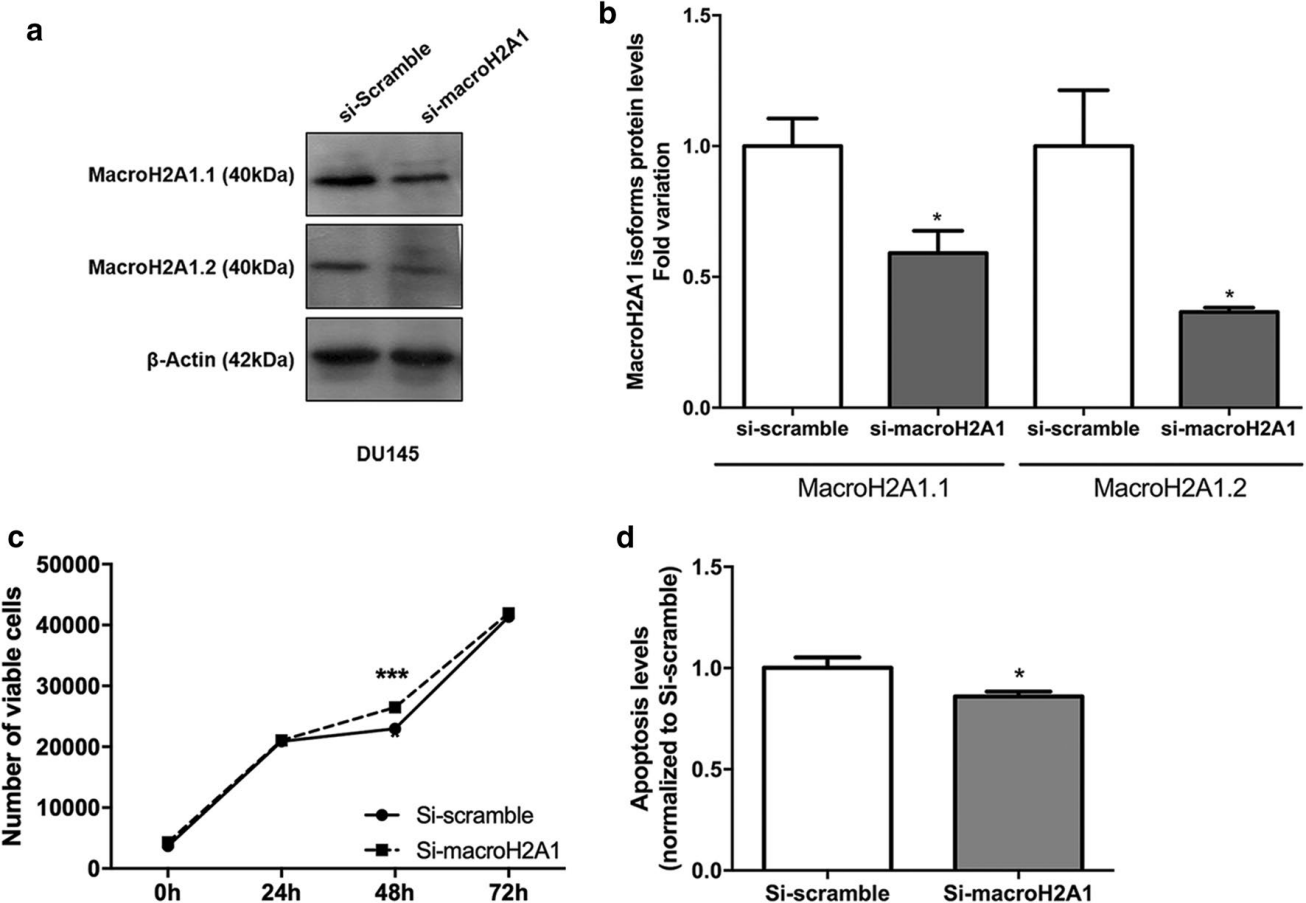
Knockdown of MacroH2A1 in DU145 cell line. **a** Illustrative images of MacroH2A1 isoforms protein after MacroH2A1 knockdown, carried out by si-RNA, in DU145 prostate cancer cell line at 72 h’ post-transfection, obtained by Western-Blot. **b** Fold variation of MacroH2A1.1 and MacroH2A1.2 protein levels in MacroH2A1-knockdown DU145 cell line, in comparison to si-scramble DU145 cell line (mean ± SD, n = 3), assessed by western-blot. Unpaired t test: *p < 0.05. Impact of MacroH2A1 knockdown in cell viability (**c**) and apoptosis levels (**d**) after 72 h (mean ± SD, n = 3). Mann–Whitney U-test: *p < 0.05, ***p < 0.001

To uncover the biological role of MacroH2A1, DU145 cells were knockdown for this protein (Fig. 4a, b). Although reduced protein levels of both MacroH2A1 variants (p < 0.05) was achieved, a more impressive effect was observed in MacroH2A1.2 (Fig. 4a, b). Phenotypically, increased cell viability was observed in MacroH2A1 knockdown DU145 cells at 48 h (p < 0.01) (Fig. 4c), whereas a significant decrease in apoptosis was apparent after 72 h, in the same cells (p < 0.05) (Fig. 4d).

**Fig. 5:**
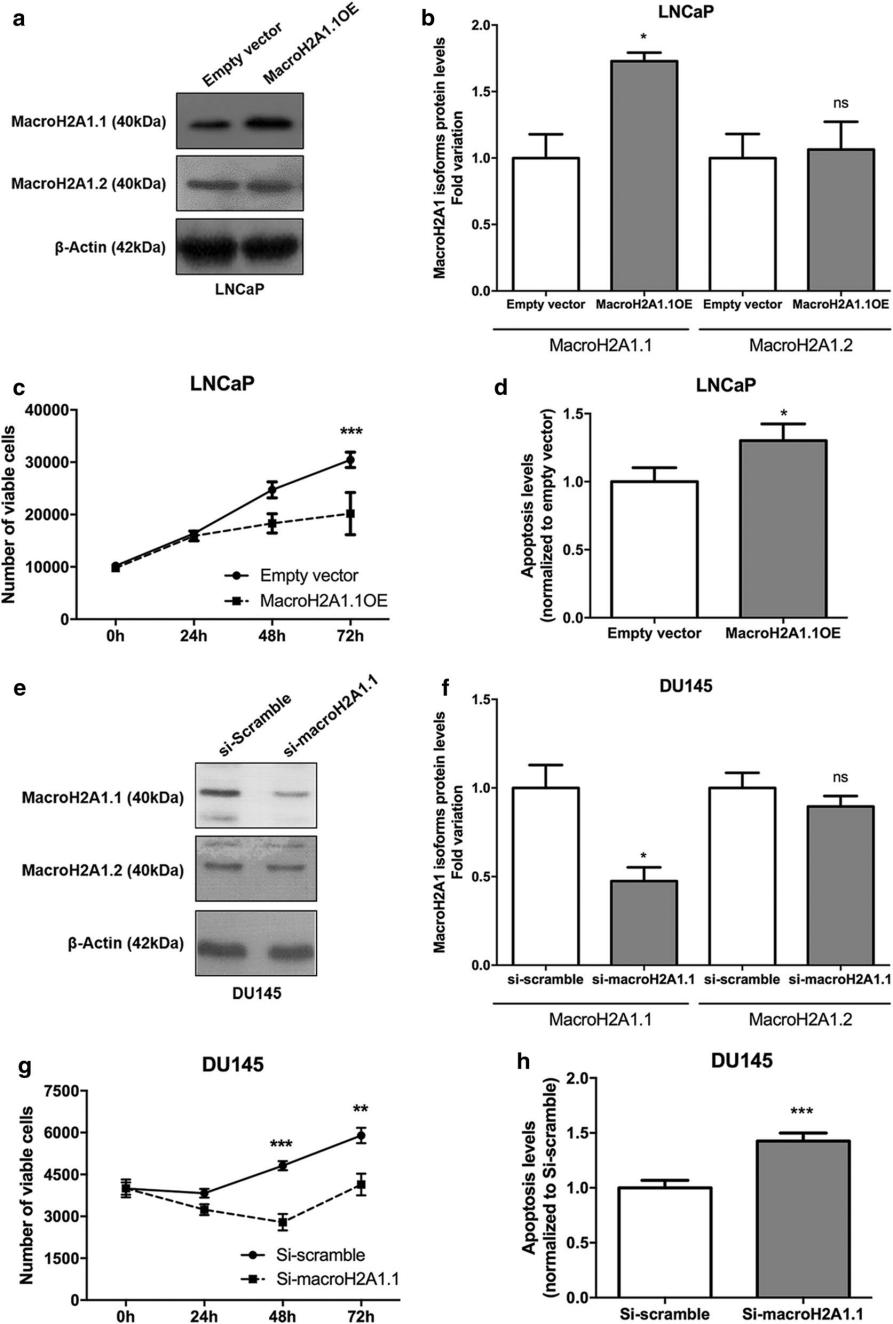
Expression modulation of MacroH2A1.1 isoform in PCa cell lines. **a** Illustrative images of MacroH2A1 isoforms protein and β-actin protein in MacroH2A1.1 transfected LNCaP cell line (LNCaP^MacroH2A1.1OE^) and transfected control cell line (LNCaP^empty vector^) at 72 h post-transfection. **b** Fold variation of MacroH2A1.1 and MacroH2A1.2 protein levels in LNCaP^MacroH2A1.1OE^, compared to LNCaP^empty vector^ (mean ± SD, n = 3). Unpaired t test: *p < 0.05, ns = not significant. Impact of MacroH2A1.1 overexpression in cell viability (**c**) and apoptosis levels (**d**) after 72 h (mean ± SD, n = 3). Mann–Whitney U-test: *p < 0.05, ***p < 0.001. **e** Illustrative images of MacroH2A1 isoforms protein after MacroH2A1.1 knockdown, carried out by si-RNA, in DU145 prostate cancer cell line at 72 h’ post-transfection, obtained by Western-Blot. **f** Fold variation of MacroH2A1.1 and MacroH2A1.2 protein levels in MacroH2A1.1-knockdown DU145 cell line, in comparison to si-scramble DU145 cell line (mean ± SD, n = 3), assessed by western-blot. Unpaired t test: *p < 0.05. Impact of MacroH2A1.1 knockdown in cell viability (**g**) and apoptosis levels (**h**) after 72 h (mean ± SD, n = 3). Mann–Whitney U-test: *p < 0.05, **p < 0.01, ***p < 0.001

Additionally, MacroH2A1.1 isoform was ectopically overexpressed in LNCaP (cell line with the lowest levels) and specifically silenced in DU145 cells (cell line with the highest levels) (Fig. 5). Effective stable MacroH2A1.1 overexpression (MacroH2A1.1OE) (Fig. 5a, b) and silencing (si-MacroH2A1.1) (Fig. 5e, f) were confirmed at protein level. Indeed, MacroH2A1.1 protein levels almost doubled in MacroH2A1.1OE LNCaP cells (p < 0.05) and significantly associated with decreased cell viability (p < 0.001) (Fig. 5c), whereas apoptosis was significantly increased 72 h after transfection (p < 0.05) (Fig. 5d). Paradoxically, MacroH2A1.1 knockdown in DU145 also resulted in decreased cell viability (p < 0.001), with a concomitant increase in apoptosis (p < 0.001) (Fig. 5g, h, respectively).

As expected, both MacroH2A1.1 overexpressing and knockdown PCa cells did not show any effect on MacroH2A1.2 protein levels (Fig. 5a, e).

## Discussion

PCa is the most common malignancy in men and a leading cause of mortality and morbidity, worldwide. Both genetic and epigenetic disruption has been implicated in its initiation and progression. Unravel the mechanisms underlying tumor development are key to provide a more profound insight into PCa biology, which might translate into better diagnostic and prognostic tools, and the identification of novel therapeutic targets [2].

Among epigenetic mechanisms, the replacement of canonical histones has been recently implicated in tumorigenesis [7]. Indeed, the role of the two MacroH2A1 isoforms have been investigated in cancer [22–26]. In particular, MacroH2A1.1 is mostly considered a tumor suppressor, inhibiting stem cell-like properties and counteracting the functions of MacroH2A1.2 [16, 17]. Although its role has been previously investigated in other tumor models, no data is available for PCa, to the best of our knowledge. Thus, we aimed to assess the putative role of MacroH2A1 isoforms in PCa initiation and progression.

We found that MacroH2A1.1 transcript levels were downregulated in PIN and primary PCa, compared to normal prostate tissues. Notwithstanding the reduced number of normal prostate tissues included in this study, which should be construed as a limitation, our results are in line with previous observations on MacroH2A1.1 expression in other primary cancers [26, 32] and the intermediate expression levels depicted in PIN is consistent with its PCa precursor condition. However, MacroH2A1.2 expression levels did not parallel the upregulation reported for other tumors [25, 26]., as no significant differences were observed between PCa and normal tissues. Contrarily, prostate cancer precursor lesion (PIN), displayed significantly lower MacroH2A1.2 transcript levels than those of MNPT and PCa. Interestingly, it was recently demonstrated that MacroH2A1.2 attenuates osteoclastogenesis in a PCa in vitro model [33], but no direct comparisons with our results can be made as we only studied organ confined PCa.

Regarding, MacroH2A1 levels, albeit PIN displayed the lowest compared to MNPT and PCa, this was mostly due to MacroH2A1.2 downregulation. Indeed, MacroH2A1.1 expression levels were lower in PCa comparing to MNPT. Thus, our results suggest that sustained MacroH2A1.1 downregulation is associated with the emergence of the malignant phenotype in the prostate, whereas concomitant MacroH2A1.2 decreased expression might be relevant for the development of PIN lesions only, which frequently do not seem to progress into invasive carcinoma. Nevertheless, MacroH2A1 downregulation is likely to play a role along prostate tumorigenesis, suggestive of a tumor suppressive effect.

Because an inverse correlation between MacroH2A1 and H2A.Z has been suggested in previous studies [29], we assessed the transcript levels of the latter in our dataset, as well, but no correlation was found between transcripts levels of those two histone variants. Considering these findings, we hypothesized that differential expression of MacroH2A1 isoforms may be related with altered expression of its splicing regulators, specifically QKI, DDX5 and DDX17. Remarkably, in primary tumors, variations in QKI and DDX17 expression levels paralleled those of MacroH2A1.1, whereas DDX5 transcript levels followed the same pattern of MacroH2A1 and MacroH2A1.2. Statistical analysis showed a moderately positive correlation between QKI and MacroH2A1.1, as well as between DDX5 and MacroH2A1 and its isoforms. Thus, we might speculate whether variations in MacroH2A1 isoforms expression in PCa is due to altered expression of its splicing regulators, although other, yet unidentified, factors might be involved, as well. In support of this hypothesis, QKI has been considered a tumor suppressor in various cancers and frequently associated with MacroH2A1.1 downregulation [21]. Concerning DDX5 and DDX17 expression, our results are somewhat unexpected as both are considered highly homologous oncogenic RNA-helicases [34]. Nevertheless, lower expression of both DDX5 and DDX17 has been reported in in situ breast cancer, along with increased MacroH2A1.1/MacroH2A1.2 ratios [22]. Remarkably, this parallels our observations in PIN lesions, which are considered pre-invasive forms of PCa. Interestingly, DDX5, but not DDX17, affect key cellular pathways, including upregulation of AR in PCa and induction of epithelial-mesenchymal transition (EMT), a feature that is associated with tumor invasion capabilities [22, 35, 36]. Interestingly, we found significant differences in MacroH2A1.1, MacroH2A1.2 and QKI, but not DDX5 and DDX17, transcript levels between AR+ and AR− PCa cell lines. Indeed, whereas the association between MacroH2A1.1 and QKI expression found in primary PCa tissues seems to hold true for PCa cell lines, the same was not evident for DDX5 and DDX17. Importantly, at protein level, significant differences between AR+ and AR− were found only for MacroH2A1.2, suggesting that other factors are involved in expression regulation of the isoforms. Nonetheless, because our series of primary PCa only represent hormone-therapy-naïve tumors, no definitive conclusions can be made and a comparative study with castration-resistant PCa cases may further elucidate these findings.

Subsequently, we focused our attention on QKI and MacroH2A1.1 expression in a subset of cases with matched PIN and PCa tissues. Although paired lesions were found in the same gland, a direct causal link between them should not be construed. Nevertheless, this analysis might elucidate how QKI and MacroH2A1.1 expression is altered along the carcinogenic process in the prostate gland. In approximately two-thirds of these cases, both QKI and MacroH2A1.1 expression was lower in PCa samples compared to matched PIN, a finding that parallels the observed variations in the whole case series. Moreover, this result further supports a causal role for QKI downregulation in MacroH2A1.1 decreased expression along prostate tumorigenesis. Indeed, decreased QKI and MacroH2A1.1 expression levels are clearly associated with PCa, as demonstrated by its ability to discriminate cancerous from non-cancerous prostate tissues, notwithstanding the limited number of the latter samples (n = 15).

Some interesting associations between QKI and MacroH2A1.1 expression and clinicopathological parameters were depicted. Specifically, PCa with higher Gleason score (i.e., less differentiated) displayed lower QKI and MacroH2A1.1 levels. Concerning MacroH2A1.1 expression, the same has been reported for other carcinomas, being its loss associated with worse outcome in colon cancer patients [26]. Conversely, MacroH2A1.1 expression was higher in PCa patients with higher serum PSA levels at diagnosis. Eventually, a comparison based on the basic Gleason patterns (3, 4 or 5) instead of the Gleason score might be considered more biologically relevant. However, we should emphasize that statistical associations were evaluated between clinicopathological parameters and molecular data, and, in this regard, the meaningful comparison is among Gleason scores and not Gleason grades, as the latter are only used as a basis for Gleason score [30]. Moreover, Gleason grade 3, 4 or 5 tumor areas are frequently intermingled and are not easily discriminated in tumors with mixed grades. Thus, an attempt to selectively collect those areas would most likely result in “contamination” and consequent analysis bias. On the other hand, if only 3 + 3, 4 + 4 and 5 + 5 tumors would be analyzed, most of tumors, which are Gleason score 7 (3 + 4 or 4 + 3) would be excluded. Moreover, the procedure used for selectively identifying the index tumor assured that the tumor area selected for molecular analyses was representative of the index tumor. Although the association with the Gleason score seems intuitive as higher scores correspond to less differentiated and more aggressive PCa, the association with serum PSA levels, on the contrary, is almost counterintuitive. Nevertheless, it should be recalled that cells from less differentiated PCa produce less PSA, which might have a negative impact on global serum PSA levels notwithstanding heavy disease burden and corresponding poor outcome [37]. Overall, these findings suggest that lower QKI and MacroH2A1.1 expression levels might be associated with worse PCa-related survival, a hypothesis that follows the same reported for other cancer models, but which requires further investigation in a larger cohort of PCa patients.

To further understand MacroH2A1 biological role in PCa, the phenotypic effect of MacroH2A1 silencing was evaluated in DU145 PCa cell line, which resulted in significantly increased cell viability, paralleling previous observations in bladder cancer cells [17, 32]. Because in primary PCa MacroH2A1.1 levels predominate over those of MacroH2A1.2, we further assessed the phenotypic impact of its overexpression and silencing. In LNCaP cells, MacroH2A1.1 forced expression significantly decreased cell viability and increased apoptosis, which is accordance with a putative tumor suppressive role [25, 26]. However, in DU145 cells, MacroH2A1.1 knockdown disclosed opposite results to those of MacroH2A1 knockdown in the same cells (which affected both isoforms, MacroH2A1.1 and MacroH2A1.2). Interestingly, it was suggested that MacroH2A1 isoforms may have different effects [18, 27], since reduced MacroH2A1.1 expression has been associated with a more aggressive phenotype [25, 26], whereas increased MacroH2A1.1 correlated with poor prognosis in triple-negative breast cancer patients [23]. Thus, its putative role might be tumor model-dependent, since MacroH2A1.1 might have an activating or repressive function depending on external cellular signals [38]. This may explain, at the least partially, our paradoxical finding in MacroH2A1.1 silenced DU145 cells. Furthermore, the modest silencing achieved for MacroH2A1.1 (only about 50%), might have also contributed to that result.

## Conclusions

In conclusion, this study is the first to report variations in expression of MacroH2A1 and its isoforms in prostate tissues, encompassing morphologically normal and neoplastic (both pre-invasive and invasive) lesions. Globally, we found that MacroH2A1.1 transcript levels gradually decrease during tumorigenesis, whereas MacroH2A1 and MacroH2A1.2 were downregulated only in PIN lesions. Interestingly, variations in MacroH2A1 are mostly affected by MacroH2A1.2 isoform and these alterations are associated with altered expression of splicing regulators, specifically QKI for MacroH2A1.1, as well as DDX5 for MacroH2A1 and isoforms ratios. Moreover, less differentiated and more aggressive PCa cases display lower QKI and MacroH2A1.1 transcripts levels, as expected for putative tumor suppressors. Finally, the attenuation of malignant phenotype of PCa cell lines after manipulation of Macro H2A1 expression, further suggest a tumor suppressor role for this histone variant, although MacroH2A1.1 and MacroH2A1.2’s role in PCa require further investigation.

## Additional files

**Additional file 1: Data S1.** To illustrate the confidence intervals for the AUC that can be obtained with these data, a simulation study has been performed.

**Additional file 2: Table S1.** Distribution of expression levels (assessed by RT-qPCR) for total MacroH2A1, splice variants and regulators among different prostate tissue samples. **Table S2.** Spearman’s ρ correlations among total MacroH2A1 and splice variants with three splicing regulators.

**Additional file 3: Fig. S1.** Scatter blots representation of transcript levels of MacroH2A1.1 and QKI in matched PIN and PCa lesions from the same patients, assessed by RT-qPCR and normalized to GUSB mRNA levels. **Fig. S2.** Correlation between MacroH2A1 and H2A.Z transcript levels in prostate cancer samples, assessed by RT-qPCR and normalized to GUSB. **Fig. S3.** Scatter blots representation of distribution of MacroH2A1.1 mRNA levels in prostate cancer tissue samples, assessed by RT-qPCR and normalized to GUSB, according to categorized ≤ 10 ng/mL and > 10 ng/mL PSA levels. **Fig. S4.** Scatter blots representation of distribution of MacroH2A1.1 and QKI mRNA levels assessed by RT-qPCR and normalized to GUSB, among Gleason scores ≤ 6, 7 (3 + 4), 7 (4 + 3) and ≥ 8 prostate cancer tissue samples. **Fig. S5.** (a) Distribution of transcript levels of total MacroH2A1, MacroH2A1.1 and MacroH2A1.2, assessed by RT-qPCR and normalized to GUSB mRNA levels, in androgen-receptor positive prostate cancer cell lines (22Rv1, LNCaP and VCaP), and in androgen-receptor negative prostate cancer cell lines (DU145 and PC-3). (b) Distribution of transcript levels of splicing regulators DDX5, DDX17 and QKI, assessed by RT-qPCR and normalized to GUSB, in androgen positive and negative prostate cancer cell lines. (c) Distribution of MacroH2A1.1 and MacroH2A1.2 protein levels, normalized to β-actin, in androgen positive and negative prostate cancer cell lines. Mann–Whitney U-test: **p < 0.01, ***p < 0.001. ns—non significant.

## Abbreviations

AR: androgen receptor
BSA: bovine serum albumin
DDX5: deadbox 5 (also known as p68)
DDX17: deadbox 17 (also known as p72)
EMT: epithelial mesenchymal transition
FFPE: formalin-fixed, paraffin embedded
GUSβ: β-glucuronidase
H3K27me3: tri-methylated histone H3 at lysine 27 (repress transcription)
MNPT: morphologic normal prostate tissue
mRNA: messenger ribonucleic acid
PCa: prostate cancer
PIN: prostate intraepithelial neoplasia
PSA: prostate-specific antigen
qRT-PCR: quantitative reverse transcriptase polymerase chain reaction
QKI: quaking, KH domain containing RNA binding
