# Supplementary material for: Histone variant MacroH2A1 is downregulated in prostate cancer and influences malignant cell phenotype
Source: Cancer Cell Int. 2019 Apr 29;19:112. doi: 10.1186/s12935-019-0835-9 (PMC6489299; doi:10.1186/s12935-019-0835-9)
Supplement: Supplementary file 1 — Additional file 1: Data S1. To illustrate the confidence intervals for the AUC that can be obtained with these data, a simulation study has been performed. [file 12935_2019_835_MOESM1_ESM.docx]

Supplementary Data 1: AUC and 95% confidence interval estimation

To illustrate the confidence intervals for the AUC that can be obtained with these data, a simulation study has been performed. First, we fitted a log-normal distribution to the gene expression in the normal tissues and another to the gene expression in tumours.

The two fitted distributions together:

Using these fitted distributions, we extracted 1000 random samples considering the same number of cases and controls as the real situation (n=197 and n=15, respectively). For each sample the AUC and the respective confidence interval range was calculated.

The mean confidence interval range obtained in the 1000 simulations was 0.101. The 25% and 75% percentiles were 0.079 and 0.119. The value obtained with the real dataset was 0.089, thus perfectly within the range of expected values.
