# Supplementary material for: Histone variant MacroH2A1 is downregulated in prostate cancer and influences malignant cell phenotype
Source: Cancer Cell Int. 2019 Apr 29;19:112. doi: 10.1186/s12935-019-0835-9 (PMC6489299; doi:10.1186/s12935-019-0835-9)
Supplement: Supplementary file 2 — Additional file 2: Table S1. Distribution of expression levels (assessed by RT-qPCR) for total MacroH2A1, splice variants and regulators among different prostate tissue samples. Table S2. Spearman’s ρ correlations among total MacroH2A1 and splice variants with three splicing regulators. [file 12935_2019_835_MOESM2_ESM.docx]

Additional File 2

Table S1: Distribution of expression levels (assessed by RT-qPCR) for total MacroH2A1, splice variants and regulators among different prostate tissue samples.

|  | **Relative Expression Mean (95% CI)** | | |
| --- | --- | --- | --- |
|  | **MNPT** | **PIN** | **PCa** |
| **MacroH2A1** | 0.54 (0.43 - 0.65) | 0.34 (0.28 - 0.39) | 0.50 (0.46 - 0.54) |
| **MacroH2A1.1** | 3.15 (2.48 - 3.82) | 2.39 (1.61 - 3.16) | 1.33 (1.21 - 1.44) |
| **MacroH2A1.2** | 0.58 (0.49 - 0.66) | 0.06 (0.04 - 0.09) | 0.59 (0.54 - 0.65) |
| **QKI** | 4.76 (3.76 - 5.76) | 4.81 (3.49 - 6.13) | 1.65 (1.44 - 1.87) |
| **DDX5** | 2.21 (1.69 - 2.73) | 0.77 (0.58 - 0.96) | 1.75 (1.58 - 1.91) |
| **DDX17** | 5.99 (4.18 - 7.80) | 2.58 (2.20 - 2.96) | 2.11 (1.90 - 2.32) |

CI – lower and upper 95% confidence interval of mean, MNPT – morphologically normal prostate tissue, PIN – prostatic intraepithelial neoplasia, PCa – prostate carcinoma,

**Table S2:** Spearman’s ρ correlations among total macroH2A1 and splice variants with three splicing regulators.

| **Genes** | **MacroH2A1** | **MacroH2A1.1** | **MacroH2A1.2** |
| --- | --- | --- | --- |
| **QKI** | ρ=0.18, p=0.92 | **ρ=0.56, p<0.001** | ρ=-0.07, p=0.25 |
| **DDX5** | **ρ=0.51, p<0.001** | **ρ=0.22, p<0.001** | **ρ=0.32, p<0.001** |
| **DDX17** | **ρ=0.30, p<0.001** | **ρ=0.41, p<0.001** | ρ=-0.03, p=0.09 |
