# Supplementary material for: Histone variant MacroH2A1 is downregulated in prostate cancer and influences malignant cell phenotype
Source: Cancer Cell Int. 2019 Apr 29;19:112. doi: 10.1186/s12935-019-0835-9 (PMC6489299; doi:10.1186/s12935-019-0835-9)
Supplement: Supplementary file 3 — Additional file 3: Fig. S1. Scatter blots representation of transcript levels of MacroH2A1.1 and QKI in matched PIN and PCa lesions from the same patients, assessed by RT-qPCR and normalized to GUSB mRNA levels. Fig. S2. Correlation between MacroH2A1 and H2A.Z transcript levels in prostate cancer samples, assessed by RT-qPCR and normalized to GUSB. Fig. S3. Scatter blots representation of distribution of MacroH2A1.1 mRNA levels in prostate cancer tissue samples, assessed by RT-qPCR and normalized to GUSB, according to categorized ≤ 10 ng/mL and > 10 ng/mL PSA levels. Fig. S4. Scatter blots representation of distribution of MacroH2A1.1 and QKI mRNA levels assessed by RT-qPCR and normalized to GUSB, among Gleason scores ≤ 6, 7 (3 + 4), 7 (4 + 3) and ≥ 8 prostate cancer tissue samples. Fig. S5. (a) Distribution of transcript levels of total MacroH2A1, MacroH2A1.1 and MacroH2A1.2, assessed by RT-qPCR and normalized to GUSB mRNA levels, in androgen-receptor positive prostate cancer cell lines (22Rv1, LNCaP and VCaP), and in androgen-receptor negative prostate cancer cell lines (DU145 and PC-3). (b) Distribution of transcript levels of splicing regulators DDX5, DDX17 and QKI, assessed by RT-qPCR and normalized to GUSB, in androgen positive and negative prostate cancer cell lines. (c) Distribution of MacroH2A1.1 and MacroH2A1.2 protein levels, normalized to β-actin, in androgen positive and negative prostate cancer cell lines. Mann–Whitney U-test: **p < 0.01, ***p < 0.001. ns—non significant. [file 12935_2019_835_MOESM3_ESM.pdf]

Additional File 3

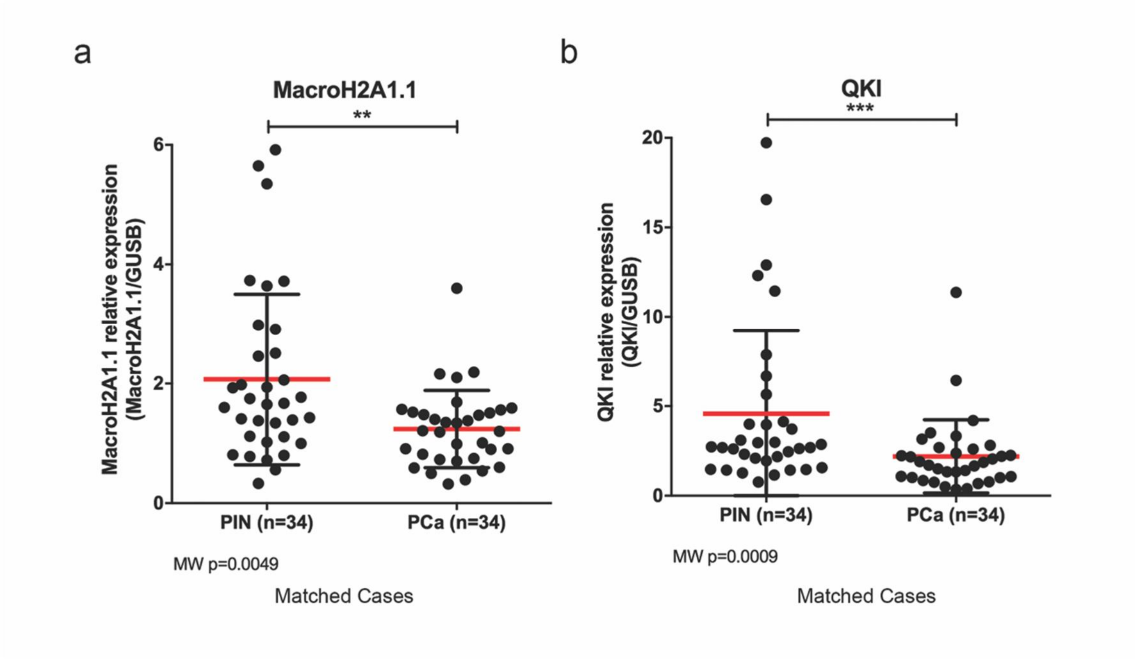

Fig S1 Transcript levels of **(a)** macroH2A1.1 and **(b)** QKI in matched PIN and PCa (n=34) lesions from the same patients, assessed by RT-qPCR and normalized to GUSB mRNA levels. Mann-Whitney U-test: **p<0.01, ***p<0.001

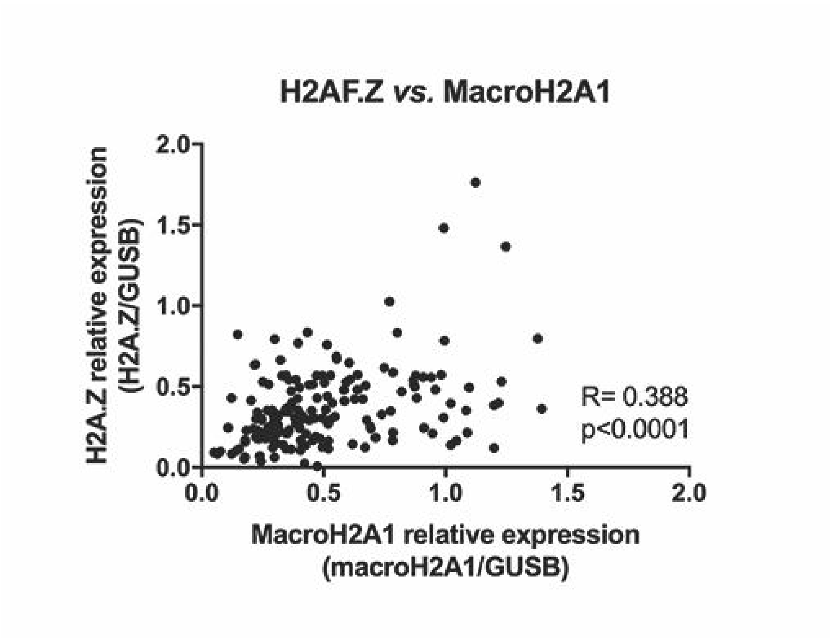

Fig S2 Correlation between macroH2A1 and H2A.Z transcript levels ($R=0.388$, $p<0.0001$) in prostate cancer samples (n=197), assessed by RT-qPCR and normalized to GUSB. (R coefficient calculated by Spearman's rank correlation)

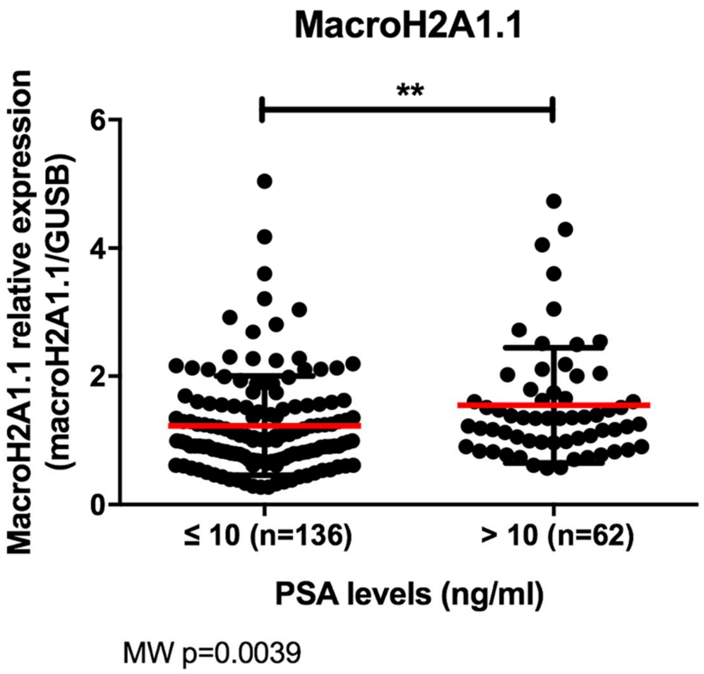

Fig S3 Distribution of macroH2A1.1 mRNA levels in prostate cancer tissue samples, assessed by RT-qPCR and normalized to GUSB, according to categorized ≤10 ng/mL (n=136) and >10 ng/mL (n=62) PSA levels . Mann-Whitney U-test: **p<0.01

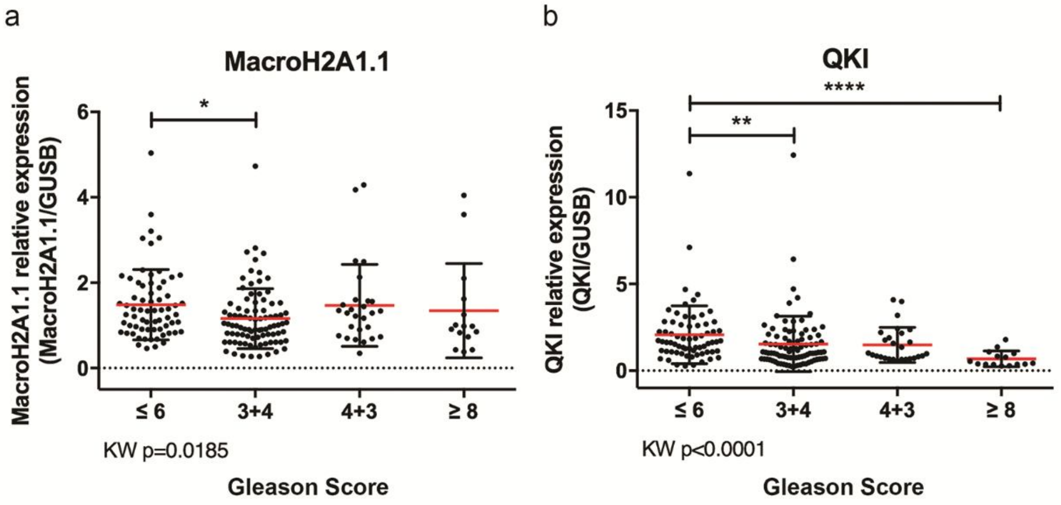

Fig S4 Distribution of **(a)** MacroH2A1.1 and **(b)** QKI mRNA levels assessed by RT-qPCR and normalized to GUSB, among Gleason scores ≤6, 7 (3+4), 7 (4+3) and ≥8 prostate cancer tissue samples. Kruskal-Wallis Test: Adjusted *p<0.05, **p<0.01, ****p<0.0001.

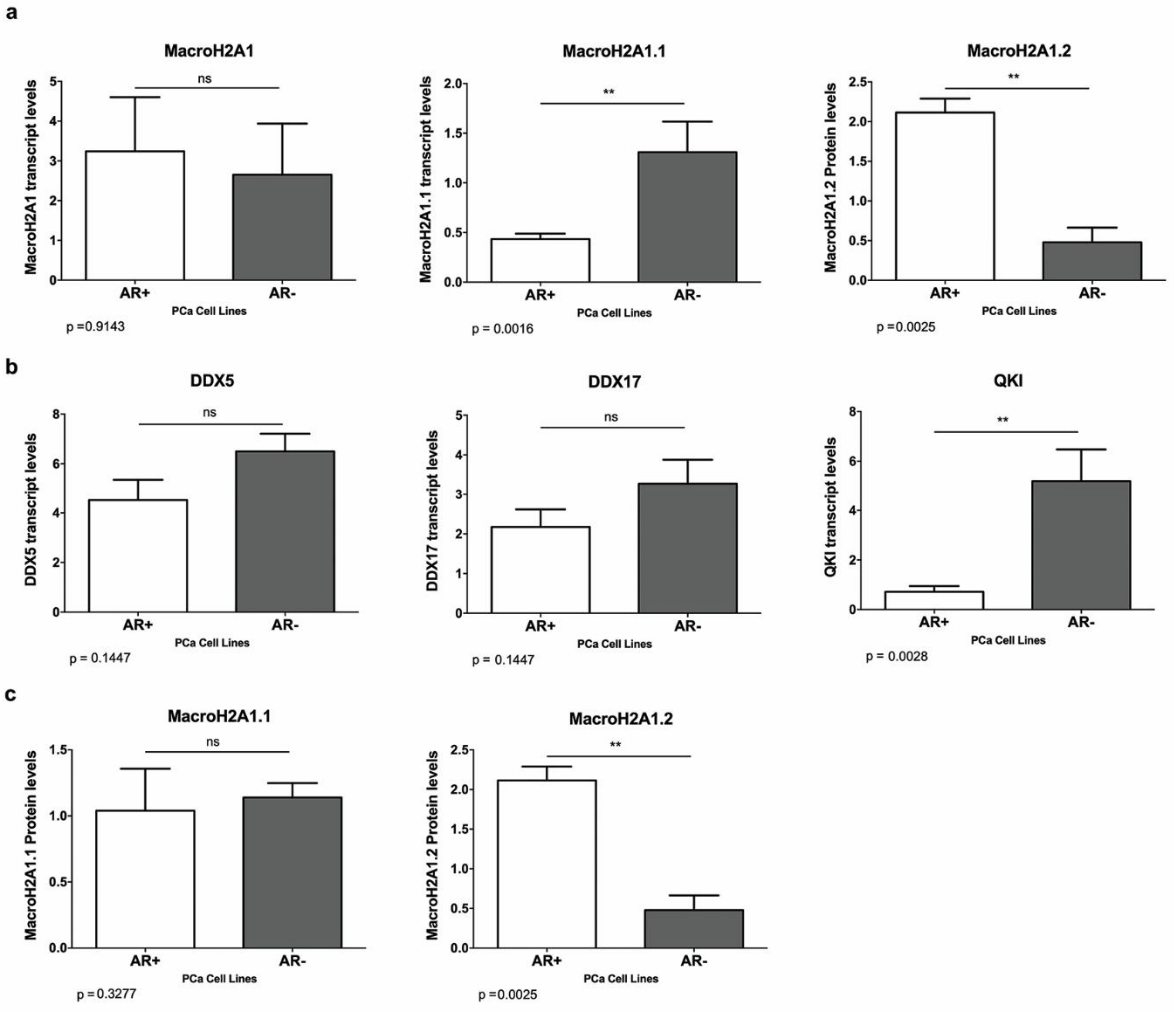

Fig S5 (a) Distribution of transcript levels of total macroH2A1, macroH2A1.1 and macroH2A1.2, assessed by RT-qPCR and normalized to GUSB mRNA levels, in androgen-receptor positive prostate cancer cell lines (22Rv1, LNCaP and VCaP), and in androgen-receptor negative prostate cancer cell lines (DU145 and PC-3). **(b)** Distribution of transcript levels of splicing regulators DDX5, DDX17 and QKI, assessed by RT-qPCR and normalized to GUSB, in androgen positive and negative prostate cancer cell lines. **(c)** Distribution of macroH2A1.1 and macroH2A1.2 protein levels, normalized to β-actin, in androgen positive and negative prostate cancer cell lines. Mann-Whitney U-test: **p<0.01, ***p<0.001. ns – non significant.
